# Book Reviews

**Published:** 1823-02

**Authors:** 


### MEDICAL AND SCIENTIFIC BIBLIOGRAPHY.

Vexat censura ...... Corvos.

Art. I.—*A Lecture, in which the Nature and Properties of Oxalic Acid are contrasted with those of Epsom Salts, and a very safe and effectual Method of preventing those fatal Consequences which have invariably followed the inadvertent Substitution of the former for the latter, is submitted to the Public; (together with the Symptoms,) and the Treatment to be adopted, when Oxalic Acid has been introduced into the Stomach, is fully investigated, and its Principles explained.* By Robert Venables, m.b. of Trinity College, Dublin.—8vo. pp. 56. Callow and Wilson, London. 1822.

This Lecture was delivered before a numerous and respectable body of the inhabitants of Henley-upon-Thames and its vicinity, to whom it is dedicated: it is, therefore, popular in its object, and very properly familiar in its execution. On this account, the author need not stand in awe of the criticisms of the candid part of his professional brethren, who certainly will receive with indulgence, and forward with zeal, any plan which appears likely to benefit the interests of humanity. Such being his object, we give the author of this little pamphlet credit for avoiding all unnecessary display of learning, and for addressing himself solely to the good sense and right feelings of his auditory. Whether the plan that he has suggested be the best that can be adopted, or whether it would be productive of all the good he anticipates, if adopted, is a question upon which more difference of opinion may exist. The subject, though a plain and simple one, is highly interesting and important, and it does not appear that any of the suggestions yet thrown out have had the effect of putting an end to the mischievous and fatal consequences which these mistakes produce. To all the methods hitherto proposed to prevent these accidents, (excepting one,) the same objection applies,—they do not go to the source of the evil: any one of them will answer the purpose, if adopted, in every instance; but the same "original sin" of carelessness which produces the first mistake is equally likely to prevent the successful adoption of the preventive measures themselves. Thus, it is said, the taste will at once detect the difference; but there are some who never can be prevailed upon to taste their medicines before they swallow them, and others who, having tasted, are incapable of retaining upon their stomach the medicine so tasted previously. Our author's plan is to apply the test of litmus or turmeric paper to this salt. After observing upon the superiority of his suggestion over that of colouring the crystals of oxalic acid, (which, besides, renders them unfit for the purposes for which they are employed,) he goes on to detail his plan, and which we shall give in his own words:

"An account of this plan (by litmus paper,) has appeared in a periodical publication; and, in order to avoid any after-charge of plagiarism, I shall take this opportunity of observing, that the article, as inserted in that publication, was written by myself. In that article I have proposed that every chemist and vender of medicines should put into each packet of those articles (Epsom salts and oxalic acid,) which he sells, a small slip of the test-paper, and that a paper of printed instructions should accompany it. But, since I have thought of applying the reddened turmeric paper to the same purpose, the litmus test-paper might accompany one description, and the turmeric test the other. The printed instructions should be concise, as thus:—'The blue colour of the accompanying test-paper is unalterable by a solution of Epsom salts; but will be immediately reddened by a solution of oxalic acid, which is poisonous.'

"If a litmus test be put up with Epsom salts, a turmeric test might accompany oxalic acid.

"The instructions for this test should be, 'The reddish brown colour of the accompanying test-paper is unalterable by a solution of Epsom salts; but will be instantly converted to a bright yellow by a solution of oxalic acid, which is poisonous.' Even if a mistake were made in putting up the tests, it would not at all affect the detection of error; because the instructions in both include the phenomena produced by the application of each of the solutions upon the test.

"It may appear superfluous, at first view, that the chemist should have the responsibility of putting up the tests with these articles, when they are so easily obtained, and every one may provide himself with the means of safety. But when it is recollected that, in the country, a number of poor persons are constantly using the sulphate of magnesia, who would not provide themselves with the means of testing it,—and, even if they should obtain the test, could not use it without instructions,—the advantages of the plan proposed will be sufficiently obvious.

"Also, if oxalic acid should happen to be lost by one individual and found by another, the advantages of the test accompanying the parcel must be very manifest. The first fatality from oxalic acid happened under such circumstances. Some person purchased a small quantity of oxalic acid, about an ounce, and in returning home, lost it from the pocket: a poor woman found it, took it home, and gave it to her husband. I need scarcely remark, that death very speedily ensued. Had a test with printed instructions, as already proposed, accompanied this parcel, it is more than probable such a fatal mistake would not have been committed."

We say nothing about the trouble and inconvenience of the plan itself, because we think with our author that this goes for nothing, in comparison with the health and lives of the community; but we think that, even if this should become the subject of a legislative enactment, that the same carelessness which will cause the one preparation to be sold instead of the other would occasion as numerous omissions of the printed directions, or perhaps a substitution of the wrong for the right one. Besides, most of the sufferers are likely to be in the lowest class of life, and unable to read the directions when they have got them; and some will not choose to apply to their more learned neighbours for assistance in a transaction which is intended to be done secretly. We are of opinion, upon the whole, that the only way to put a stop to these melancholy occurrences, is to prohibit the sale of this article in toto: we are not aware of its being used for any purpose excepting that of cleaning boot-tops, which is equally well accomplished by the dilute sulphuric acid, or in many other ways; and we do not conceive that the fine and polished appearance of all the boot-tops in Europe is to be put in comparison with the life of the poorest and meanest subject of this or any other country. We need not follow our author through the remainder of his Lecture, in which he describes the symptoms that are produced by swallowing this poison, as well as the measures to be adopted upon such unfortunate occasions. This publication will be useful, undoubtedly, as far as it goes: it will help to extend the knowledge of the distinctions between the oxalic acid and Epsom salts; and this knowledge alone, when become universal, will perhaps render any farther precaution unnecessary.

---

ART. II.—*A Treatise on the Utility of Sangui-Suction, or Leech-Bleeding, in the Treatment of a great Variety of Diseases, including the Opinions of eminent Practitioners, ancient and modern; with Instructions for the Process of Leeching; and an Appendix, delineating the characteristic Distinctions of true Leeches, with Directions for their Management and Preservation.* By REES PRICE, M.D. Surgeon, Member of the Royal College of Surgeons, &c.—12mo. pp. 152. Simpkin and Marshall, London. 1822.

THIS little work commences with a learned introduction, tracing the origin of leech-bleeding to the remotest antiquity, and concluding with a remark, which we believe to be perfectly well founded, "that leeching has never obtained in England the same free and almost unlimited extension as on the continent:" but we are not inclined to concede to Dr. Price, that our partiality for the lancet should ever be a matter of reproach, although the beneficial effects of the application of leeches may have in some instances been too much overlooked.

The mode in which Dr. Price has published his observations renders it necessary for us merely to touch upon those few points in which we either happen to differ with him in opinion, or where we think it necessary to enforce, or to notice, any important observation that he has made; for, as he has advocated the employment of leeches in no less than ninety-two different complaints, it becomes impossible for us to mention each of these individually, or to follow him step by step. We find, under the head of Fevers, our author deeply engaged in the physiological inquiry concerning the cause of inflammation, and the state of the minute arterial branches in that disease, into which we cannot at present enter, and which, perhaps, we should have passed by altogether, but for the concluding sentence, which is as follows:—"It has been supposed, and I think with some probability of truth, that leeches excite an increased action of the absorbents of the part," &c. (p. 15;) a position which we think very questionable, and the rationale of which we do not quite understand.

We shall pass by the observations of our author relating to fevers and inflammatory affections, in order to notice a passage under the head of Phrenitis, and which we consider important, because the practice there recommended is generally too much neglected in this country, and we are quite satisfied of its great utility:

"Zacutus Lusitanus speaks highly of leeching in this affection, and reports the case of a lady, who, whilst labouring under a retention of the menses, was attacked with phrenitis, as the consequence. Four leeches were fastened by their extremities to a thread, which gave the person who applied them the power of preventing their insinuating themselves beyond the part selected; and they were then placed within the vagina, very near to the uterus: a considerable quantity of blood was lost, to the complete relief of the disease. This author was not only very much attached to the practice of leeching, but he also was particularly biassed to the selection of the hemorrhoidal veins as the most proper spot for their application, in consequence of the great connexion which, he justly supposed, there exists between these veins and other parts of the body."

We are inclined, however, to look upon the practice of Doctors Duchatelet and Martinet with some little suspicion; and we must not suffer ourselves to be so swayed by the love of novelty as to be induced to neglect the more powerful means of depletion by the lancet in the inflammatory affections of the membranes of the brain, which they have described: it is probable that, had a more energetic use of the lancet been adopted in the majority of these cases, the proportion of those who recovered would have been much more considerable than it appears to have been. We grant that blood-letting is strongly recommended in the didactic portion of their work; but the detail of cases displays a very different, and far less energetic, practice to have been usually adopted.

We find nothing to notice particularly in the course of our author's remarks on the inflammatory affections of the different viscera; but we pause at page 48, in order to confirm his observation, that *metastasis of rheumatism* does often take place to the heart, not only in weak constitutions, but in some very vigorous ones; one example of which we shall have occasion to record in the next Number of this Journal. The fact is highly important, and, though noticed by various authors, it does not appear to be so generally kept in mind as it deserves to be; because, when it occurs, nothing short of the most vigorous means can save the patient.

We much question the propriety of leech-bleeding in erysipelas: with us, that disease generally assumes a character forbidding the abstraction of blood. There are certainly numerous exceptions; but, as a general precept, we should be inclined to say, the application of leeches in erysipelas is neither good nor safe practice.

The same caution applies to the employment of leeches in scrofulous glandular enlargements about the neck, &c. We are firmly persuaded that suppuration is as frequently induced by the use of all the active measures usually employed to disperse these tumors, as not; the irritation produced by the leech-bites often causing an action in the part, which had long remained indolent, and which, when once excited, it is very difficult to control, or direct to the end proposed,—that of dispersing the tumor.

On the subject of Tetanus, our author is rather more diffuse than usual, and he advocates warmly the depleting system for the cure of this affection, in opposition to the plan, more usually in vogue, of administering wine, opium, and other stimuli. Both modes of treatment have been very extensively tried, and both as frequently have been found to fail. We conceive that the age, temperament, situation, and habits of life of each individual patient, must be taken into account before the practitioner can venture to direct what line of conduct it is prudent or judicious to adopt. In traumatic tetanus, the depletory system we conceive to be most likely to succeed; in idiopathic, as the cause is obscure, either plan may have its advocates, and each may be enabled to produce some instances of success.

Our author very judiciously recommends sangui-suction in certain conditions of the menstrual discharge, (p. 77;) but, as most practitioners acknowledge their utility in these affections, we need do no more than mention this subject generally.

The employment of leeches in infantile diseases is so generally resorted to, and their efficacy so universally acknowledged, that we shall pass over this part of Dr. Price's work without making any particular reference. In general, his remarks are judicious.

On Gonorrhœa we have a few words to say. Our author recommends, when the inflammatory affection is very severe, four, six, or eight leeches to be applied along the under part of the penis, and over the perinæum, (p. 93.) We have so frequently had occasion to see œdematous swellings of the prepuce succeed to the application of leeches on the penis, that we infinitely prefer opening the vein on the dorsum penis, or taking away blood from the perinæum by cupping. In those who have long prepuces, these *crystallines* sometimes become very large: they are extremely alarming to the patient, and, though leading to no unpleasant result, are often many days in subsiding.

Having finished the consideration of the principal diseases in which leeching is indicated, we come to the mode of performing the operation, the first paragraph of which we shall transcribe:

"The part selected for their application being cleared of hairs (if there be any,) by a razor, and having been washed with plain soap and water, and again with water alone, should be lightly moistened with milk, or, what is still better, with a little porter: the latter is in frequent use by the lower class, and amongst them I first discovered it. I have found it successful in luring the leech to fix itself, when, after the most tedious and sedulous endeavours, every other means had failed."

Leeches should not be much handled prior to their application. If the weather be cold, they are liable to become torpid, and should be warmed by breathing on them.

Leeches may be applied by means of a wine or cupping-glass. It is useful, in either case, to put a piece of stiff paper to the bottom of the glass, to which they are otherwise apt to retire; or, if they are required on a particular spot, a small glass tube, four or five inches long, and just large enough to admit the body of the leech, may be employed. If the leech remains torpid during the process of sangui-suction, a drop or two of cold water sprinkled on it will arouse it again into action. The best methods of suppressing hæmorrhage, which sometimes succeeds to their application, and is occasionally very troublesome, when all the simple means, such as felt, starch, puff-ball, &c. have failed, are to apply the spirits of turpentine, or the muriated tincture of iron; or, in failure of these, to insert a piece of lunar caustic (the point being scraped very fine,) into the puncture; or, lastly, a weak solution of tartarized antimony has lately been recommended as very efficacious. Dr. Price says that, in order to preserve the leeches, they should be suffered to retain the blood, and merely be thrown into a jar of fresh water. In the course of a month or two, they will become firm and healthy, and able to perform their functions again; but, if the time cannot be spared for their recovery, he prefers what is called *stripping them,* to making them disgorge the blood by means of salt. It seems that, if the blood is carefully squeezed from the leech, it is capable of resuming its functions immediately.

We must now take our leave of Dr. Price. We have extracted from his work a sufficient quantity of matter to show that we have derived pleasure from its perusal. It contains many hints relative to leech-bleeding not generally known, and which cannot fail of being extremely useful to the profession at large. An appendix to the work contains the natural history of the leech, its diseases, mode of preservation, &c.

---

ART. III.—***Observatio de Affectibus Morbosis Virginis Haviniensis cui Plurimæ acus e variis Corporis partibus excisæ et extractæ sunt.*** Auctore J. D. HERHOLDT, Medicinæ Doctore et Professore, protomedico Nosocomii Regii Fridericiani, Auctore Medicinæ Militaris Nauticæ, Equite ordinis Danebrogici, Membro plurium Societatum Doctarum.—pp. 44. Haviniæ, 1822.

WE laid before our readers, a few months ago, the history of this very singular case, which was communicated to us by Dr. OTTO, of Copenhagen. Since that time we have received the original account published by Dr. HERHOLDT, under whose care the subject of his observations was placed; and we find the statement we formerly published so correct that we must beg leave to refer to it. At present we shall only extract from the work before us two tables, the first showing the total number of needles removed from particular parts, and the second giving the order of their extraction with regard to time. It is worthy of remark, that Dr. Herholdt gives the names of thirty-six well-known medical men in Copenhagen, who have seen the girl, and eighteen of whom were present at the removal of some of the needles. There are limits to unbelief, and, to doubt the circumstance of an immense number of needles having been extracted, would be absurd. Whether these are to be regarded as the cause of all the suffering, is quite another question; and we leave it to the discretion of our readers to form their own opinion of the pathology of the case.

TABLE I.

*Showing the Number of Needles extracted from each Region.*

| *The Region.* | *Number of Needles.* |
|---|---|
| From the left breast | 22 |
| Between the breasts | 14 |
| From the epigastric region | 41 |
| Left hypochondriac region | 19 |
| Right hypochondriac region | 20 |
| Umbilical region | 31 |
| Left lumbar region | 39 |

| *The Region.* | *Number of Needles.* |
|---|---|
| Right lumbar region | 17 |
| Hypogastric region | 14 |
| Left iliac region | 27 |
| Right iliac region | 23 |
| Left thigh | 3 |
| Right thigh | 1 |
| Between the scapulæ | 1 |
| Beneath the right scapula | 1 |
| Total | 273 |

TABLE II.

*Showing the Chronological Order in which the Needles were extracted.*

| *Time.* | *Number.* | *Region.* |
|---|---|---|
| February 12, 1819, | 1 | From the hypogastric region. |
| 15, | 1 | ——— left lumbar region. |
| March 1, | 2 | ——— left lumbar region. |
| April 6, | 1 | ——— left lumbar region. |
| From May 8th to 26th, | 3 | ——— left lumbar region. |
| | 7 | ——— right iliac region. |
| | 4 | ——— left iliac region. |
| | 3 | ——— hypogastric region. |
| October 3, | 4 | ——— epigastric region. |
| 4, | 7 | ——— left lumbar region. |
| From October 6th to December 18th, | 8 | ——— umbilical region. |
| | 17 | ——— left lumbar region. |
| | 11 | ——— left iliac region. |
| | 12 | ——— right hypochondriac region. |
| | 5 | ——— sternum, between the mammæ. |
| | 4 | ——— left hypochondriac region. |
| From January 26th to February 8th, 1820, | 6 | ——— umbilical region. |
| | 12 | ——— epigastriac region. |
| | 6 | ——— right iliac region. |
| | 8 | ——— left lumbar region. |
| From February 26th to March 14th, | 11 | ——— epigastric region. |
| | 5 | ——— left hypochondriac region. |
| From April 8th to the 28th, | 8 | ——— right lumbar region. |
| | 10 | ——— left hypochondriac region. |
| | 8 | ——— right hypochondriac region. |
| | 9 | ——— between the mammæ. |
| From May 18th to June 15th, | 17 | ——— umbilical region. |
| | 12 | ——— left iliac region. |
| From July 3d to 20th, | 10 | ——— right iliac region. |
| | 14 | ——— epigastric region. |
| | 9 | ——— right lumbar region. |
| | 10 | ——— hypogastic region. |
| From July 30th to August 10th, | 3 | ——— left thigh. |
| | 1 | ——— between the scapulæ. |
| | 1 | ——— right thigh. |
| | 1 | ——— below the right scapula. |
| | 22 | ——— left breast. |
| Total | 273 | |

