# Erratum

**Published:** 1823-03

**Authors:** 


### ERRATUM.

In the bibliographical account of Dr. PRICE's book, in our last Number, a few lines were inadvertently omitted, from having been written on a detached slip of paper. The omission precedes the allusion to Drs. Duchatelet and Martinet.

